# Resilient coping in the general population: standardization of the brief resilient coping scale (BRCS)

**DOI:** 10.1186/s12955-017-0822-6

**Published:** 2017-12-28

**Authors:** Rüya-Daniela Kocalevent, Markus Zenger, Andreas Hinz, Burghard Klapp, Elmar Brähler

**Affiliations:** 1 Institute and Policlinic for Medical Psychology, University Medical Center Hamburg-Eppendorf, Martinistr. 52, W26, 20246 Hamburg, Germany; 2 Department of General Practice/Primary Care, University Medical Center Hamburg-Eppendorf, Martinistr. 52, W26, 20246 Hamburg, Germany; 3 Faculty of Applied Human Studies, University of Applied Sciences Magdeburg and Stendal, Breitscheidstraße 2, 39114 Magdeburg, Stendal Germany; 4 Department of Medical Psychology and Medical Sociology, University of Leipzig, Ph.-Rosenthal-Str, 55 04103 Leipzig, Germany; 5 Department of Psychosomatic Medicine, Charité University Medicine, Berlin, Germany; 6 Department of Psychosomatic Medicine and Psychotherapy, Universal Medical Center Mainz, Mainz, Rhinland-Palatinate Germany; 7 Integrated Research and Treatment Center (IFB) Adiposity Diseases - Behavioral Medicine, Medical Psychology and Medical Sociology, University of Leipzig Medical Center, Leipzig, Germany

**Keywords:** Resilience, Coping, Normative data, Brcs, General population

## Abstract

**Background:**

There has been a marked tendency for researchers, clinicians, and policy makers to shift their focus from risk to resilience. This should be assessed by comparing the outcome to a context specific reference group.

The objectives of the study were to generate normative data for the BRCS for different age groups for men and women and to further investigate the construct validity and factor structure in a general population.

**Methods:**

Nationally representative face-to face household surveys were conducted in Germany in 2013 (*n* = 2508).

**Results:**

Normative data for the BRCS were generated for men and women (53.2% female) and different age levels (mean age (SD) of 49.7 (18.0) years). Men had significantly higher mean scores compared with women (14.9 [SD = 3.2] vs. 14.6 [SD = 3.1]). The results of the EFA and CFA clearly indicate a unidimensional solution with one factor. Furthermore, the invariance of the one-factor model was tested for the whole sample across gender and six age groups.

**Conclusions:**

The normative data provide a framework for the interpretation and comparisons of resilience with other populations.

**Electronic supplementary material:**

The online version of this article (10.1186/s12955-017-0822-6) contains supplementary material, which is available to authorized users.

## Background

Most definitions of resilience emphasize two elements as crucial [1–3]. First, an input perspective: the (subjective) exposure to risk and adverse circumstances, which can vary from moderate to extreme risks environments. The second element of a resilience definition is in respect to an outcome perspective, studying whether coping mechanisms lead to outcomes within or above the expected range. According to Rutter, the concept of resilience has to be considered on the basis of evidence of risk and protection [4]. Particularly during the last two decades, there has been a marked tendency for researchers, clinicians, and policy makers to shift their focus from risk to resilience, whereby resilience represents the interaction between risk factors (vulnerability) and resources (protection) [5]. This should be assessed by comparing the outcome to a context specific reference group (e.g. same age group, social and cultural context, etc.) [6].

The Brief Resilient Coping Scale (BRCS) is a 4-item measure designed to capture tendencies to cope with stress in a highly adaptive manner [7]. The BRCS has adequate internal consistency (*r* = .76) and test-retest reliability (*r* = .71). Convergent validity of the scale is demonstrated by predictable correlations with measures of personal coping resources (e.g., optimism, helplessness, self-efficacy), pain coping behaviors, and psychological well-being [7]. So far, the BRCS was used in specific samples, such as medical students [8], in patients with systemic lupus [9], the U.S. military [10], or nursing students [11]. The BRCS showed to be a suitable one-dimensional scale for measuring resilience in patients with systemic lupus [9] and demonstrated psychometric robustness adequate for continued use in older populations [12]. Lopez-Pina et al. suggested that the BRCS might be useful for clinicians to obtain information concerning the degree of resilience that each patient has, allowing individuals with low resilience to be identified who need interventions aimed at developing coping skills [9]. Medical students reported higher scores of resilient coping compared to validation samples and results in other studies [8]. The relationship between coping and resilience among U.S. military active service members and veterans, was investigated in order to identify the coping strategies used by those considered most resilient, and to discuss coping and resilience as they relate to the workplace [10]. The study identified resilient coping strategies of the U.S. service members and veterans who had less high resilience scores.

One recent study confirmed the buffering effect of resilience in a representative sample in the German general population [13]. High trait resilient subjects showed less distress and somatoform symptoms despite reported childhood adversities in comparison to those with low resilient coping abilities.

The normative data of the underlying study may provide a framework for the interpretation and comparisons of resilient coping with different other populations. The objectives of the present study were hence to generate normative data for the BRCS for different age groups for men and women and to further investigate the construct validity and factor structure in a general population.

## Methods

### Study sample

A nationwide survey, representative of the German general population, was conducted with the assistance of an institute specialized for demographic research, USUMA, Berlin; https://www.adm-ev.de/index.php?id=76&L=1 following the ADM-Sampling-System (F2F):

The ADM-Sampling-System (F2F) is designed as an area sample covering all populated areas of Germany. It is based on Germany’s topology, organized by states, counties and communities, the statistical areas within communities described by public data, and the geographical data created for traffic navigation systems. Combining these data, the area sample is made up of about 53,000 areas, each containing at least 350 but on average about 700 private households. Prior to sampling, the areas are first regionally stratified according to counties and so-called BIK types1) resulting in some 1500 strata. Based on this stratification, 128 “nets” are extracted containing 210 areas in former West Germany and 48 in former East Germany. These 258 sampling points (= areas) are drawn proportionally to the distribution of private households. For optimal utilization of the stratified sampling frame, sampling is done using the method for random allocation developed by L. H. Cox.2) The key advantage of this method is that it leads to stratified samples without any accumulation of rounding effects. As one area is drawn for one net only and rounding effects are minimized by the Cox allocation, any selected net may be combined with any other selected net, without issues such as differing selection probabilities or too high rounding differences arising. The ADM-Sampling- System (F2F) provides member agencies with as many nets as they need to carry out their surveys. In the second step, and where necessary in the third step too, the private households (2nd step) and within them the individuals (3rd step) to be polled, are selected randomly using systematic selection methods with a random start. Such methods are known as “random walk”, “address listing with random selection”, “Kish tableau”, “next/last birthday” and others. (These two steps are performed by the agencies themselves).

Since the sampling is done randomly in all three steps (area sampling, household selection, selection of target persons), this method for face-to-face surveys is based entirely on random sampling. Therefore surveys based on this ADM-Sampling-System (F2F) fully meet the scientific requirements regarding randomization based on statistical theory.

The ethics committee of the University of Leipzig approved the study. All adult participants provided their written informed consent to participate in this study and the data to be published. Also, written informed consent from the next of kin, caretakers, or guardians on behalf of the minors/children enrolled in the study was obtained. These consent procedures were approved by the ethics committee.

The basic population for the data collection is made up of the German population aged at least 14 years and living in private households in 2006 (*N* = 2508).

The survey was carried out by professional interviewers. Within each wave, from the demographic consultation company (USUMA, Berlin), a representative sample of the German population aged 14 years or older was approached using 258 sample points. Addresses were selected according to the random route procedure. Two callbacks had to be without success before an address was considered a failure. The households and members of these households were selected via random-route procedure. This randomized sampling procedure consisted of sample points, household, and persons in the last stage. Target households within the sample points were determined using the random-route procedure: choosing sample point areas within Germany, randomly choosing households within these areas, and randomly choosing target persons within these households. There was only one person chosen within each household. The study participants were asked to fill in a set of several questionnaires on mental and physical health, including the BRCS within a face-to-face interview (Additional file 1).

### Instrument

#### Resilient coping (BRCS)

The BRCS has adequate internal consistency (α=.76) and test-retest reliability (*r* = .71). Resilient coping is conceptualized as to cope with stress in a highly adaptive manner, using a 5-point Likert scale “from ‘1’ = *describes me not at all* to ‘5’ = *describes me very well*”. The items are the following:

1. *I look for creative ways to alter difficult situations*
2. *Regardless of what happens to me, I believe I can control my reaction to it*
3. *I believe that I can grow in positive ways by dealing with difficult situations*
4. *I actively look for ways to replace the losses I encounter in life*

The sum score varies between 4 to 20. Further information on the German Version of the BRCS was reported elsewhere [14].

### Statistical analysis

As measure of the test’s reliability, Cronbach’s alpha was calculated. Effect sizes were calculated according to Hedges & Olkin [15]. In the socioeconomic categories *gender, cohabitation, and unemployment* the first subgroup (e.g. male) was used as a reference group to compute effect sizes. In the socioeconomic categories *age, marital status, education, and income* with more than two subgroups, the total sample SD was used to compute effect sizes instead of a pooled SD to put values on a comparable metric.

To determine the number of factors in the BRCS, an exploratory factor analysis (EFA) was conducted, followed by a confirmatory factor analysis (CFA). For this purpose the study population was split into two nearly equal sized random samples (N 1_EFA_ = 1.267; N 2_CFA_ = 1.234) in a first step, followed by EFA and CFA with separate samples.

For the EFA the principal axis factors method was applied and a total of four different indicators were used to identify the factor structure of the BRCS: Kaiser Guttman criterion, scree-plot, Velicer’s minimum average partial (MAP) test [16], and Horn’s parallel analysis (PA; Horn, 1965). The MAP test is based on the averaged partial correlations of the variables under study after extracting the effect of the factors successively in order to their eigenvalue. In each step the average squared partial correlations between the items are computed, and the number of factors to retain is determined by the step that resulted in the lowest average squared partial correlation [17]. PA focuses on extracting Eigenvalues from random data sets that have the same number of variables and cases compared to the original raw data. This procedure is based on the idea that factors of real data should have larger eigenvalues that those extracted from random data. Therefore, only those factors should be retained in the real data whose eigenvalues are greater than those of the random data [17].

Consequently, the factorial structure of the BRCS was tested using CFA, calculated with AMOS© 23, to compute details on the model fit and to test the invariance of the model across gender and age. For this purpose covariance matrices were used, and each model was estimated with the maximum likelihood method approach. The fit of the model was evaluated on the basis of the following model fit indices: χ^2^; the comparative-fit-index (CFI); standardized root mean square residual (SRMR); the root mean square error of approximation (RMSEA); the Tucker-Lewis Index (TLI) and the Goodness-of-Fit Index (GFI). For a good model fit, the ratio CMIN/DF should be as small as possible [18]; values of TLI, CFI and GFI close to 0.95 or higher are indicative of a good model fit. Furthermore, RMSEA should be less than 0.08, and SRMR should be 0.05 or smaller.

Additionally, further analyses were conducted to test the invariance of the model across gender and age using multi-group CFA. This is an important statistical condition before means of different subgroups can be compared with each other [19]. After testing the factorial structure in each subgroup, measurement invariance was tested in three steps using first the configural model (no constraints), followed by a metric invariant model (item loadings constrained to be equal across groups), and a scalar invariant model (item loadings and item intercepts simultaneously constrained to be equal across groups). Due to the hierarchy of these nested and increasingly restrictive models, the models were then compared to each other. Since the χ2 statistic has often been criticized for its sensitivity to sample size, we focused mainly on the difference ΔCFI. Values equal to or smaller than .01 indicate the invariance of the model [20]. Further, to avoid the problem of selecting a marker variable that is potentially not invariant, the variance of the latent variable was fixed to 1.0 (and the mean was fixed to 0.0) for scaling purposes [21].

The percentiles were calculated according to the following formula [22]: percentile rank = 100* (m + 0.5 k)/N, where m is the number of members of the sample obtaining a score lower than the score of interest, k is the number obtaining the score of interest, and N is the overall normative sample size.

## Results

### Sample characteristics

Of the 4386 addresses selected, 4360 proved valid. A total of 2508 persons agreed to participate, provided verbal informed consent, and completed the study questionnaires. The response rate among those individuals who were asked to participate by the interviewers was 57.5%. The main reasons for non-participation (42.1%) were: the general information request was refused (13.6%), the interview request was refused (12.4%), or there was no one at home for three times in a row (12.9%), or other reasons, e.g. illness, vacation etc. (3.2%).

There were significant gender, age, marital status, education level, employment status, and income effects in the general population associated with a higher BRCS score. Yet, as noted in Table 1, the calculated effect sizes were small for all sociodemographic groups.

**Table 1:** Demographic characteristics of the study sample and associations with BRCS scores

	N (%)	BRCS M (S.D.)	Group differences *P* value	Cohen’s *d*, effect-size^a^
Gender			*P* < .05	*d* = 0.09
Male	1174 (46.8)	**14.9** ^b^ **(3.3)**		
Female	1334 (53.2)	14.6 (3.1)		
Age group, yr.			*P* < .001	*d* = 0.03
14–24	257 (10.2)	14.4 (3.3)		
25–34	360 (14.4)	14.9 (3.2)		
35–44	382 (15.2)	15.1 (3.3)		
45–54	445 (17.7)	15.0 (3.0)		
55–64	454 (18.1)	**15.2 (2.9)**		
65–74	381 (15.2)	14.4 (3.2)		
≥ 75	229 (9.1)	13.4 (3.3)		
Cohabitation			*P* < .001	*d* = 0.01
Yes	1315 (52.4)	**15,0 (3.0)**		
No	1193 (47.6)	14.4 (3.3)		
Marital Status			*P* < .001	*d* = 0.02
Married	1112 (4.3)	15.0 (3.0)		
Separated	64 (2.6)	**15.7 (3.2)**		
Single	705 (28.1)	14.7 (3.3)		
Divorced	351 (14.0)	14.6 (3.3)		
Widowed	276 (11.0)	13.7 (3.2)		
Education			*P* < .000	*d* = 0.04
None	67 (2.7)	12.7 (4.0)		
High School	1810 (72,5)	14.6 (3.2)		
College	323 (12,9)	15.4 (3.0)		
University	220 (8.8)	**16.2 (2.6)**		
Currently Student	78 (3,1)	13.8 (3.5)		
Unemployment			*P* < .05	*d* = 0.02
Yes	142 (5.7)	14,1 (3.5)		
No	2366 (94.3)	**14.8 (3.2)**		
Net household income			*P* < .001	*d* = 0.02
< 1250 €/month	593 (23.6)	14.1 (3.5)		
1250- < 2500 €/month	1146 (45.7)	14.7 (3.1)		
≥ 2500 €/month	769 (30.7)	**15.3 (3.1)**		

^a^Cohen’s defined effect sizes as follows: “small, d = .2”, “medium, d = .5”, and “large, d = .8”

^b^Bolded means in the table represent the subgroups with the highest mean score

### Factor analyses

The results of the EFA clearly indicate a unidimensional solution with one factor. The Kaiser Guttman criterion (eigenvalues > 1) resulted in one factor with an eigenvalue of 2.48, accounting for 62% of the variance. Factor loadings of the unrotated component matrix varied between .72 and .85 (Table 2).

**Table 2:** Factor loadings derived from EFA using principal axis factors method (unroated component matrix) *N* = 2508

Item	Unrotated solution
	Component 1
*I look for creative ways to alter difficult situations*	.816
*Regardless of what happens to me, I believe I can control my* *reaction to it*	.758
*I believe that I can grow in positive ways by dealing with* *difficult situations*	.853
*I actively look for ways to replace the losses I encounter in life*	.718

The visual evaluation method of the scree-plot indicated one factor, too (figure not shown). Additionally, MAP test (Velicer, 1976) and PA (Horn, 1965) indicated a unidimensional scale structure. Results are shown in Table 3.

**Table 3:** Results of minimum average partial test and parallel analysis (*N* = 2508)

Factors	MAP test	PA Eigenvalues	
	Average squared partial correlations	Raw data	Random data^a^
0	.2482		
1	.1165	2.4827	1.0964
2	.3944	.6766	1.0408
3	1	.4580	1.0004
4		.3827	.9678

*MAP* Velicer’s minimum average partial test, *PA* parallel analysis

^a^Eigenvalues corresponding to the 95th percentile of the distribution of random data eigenvalues, which are based on 1000 random data sets

Consequently, a unidimensional model was tested in the CFA. Results are shown in Table 4. Results of CFAs clearly indicate a good model fit with one exception: the χ^2^-Test was significant with *p* = .011. Since the χ^2^-Test is sensitive to sample, it would always lead to a rejection of a model examined in big samples like. Thus, regarding all fit indices synoptically, the unidimensional model tested here fits the empirical data very well. Factor loadings ranged between .61 and .80.

**Table 4:** Summary of fit indices of the unidimensional factor model

	χ ^2^ (df)	CFI	SRMR	RMSEA (CI)	TLI	GFI
One-factor model	8.987 (2)	.995	.015	.053 (.022–.091)	.984	.996

*df* degrees of freedom, *CMIN/DF* minimum discrepancy, divided by its degrees of freedom, *CFI* comparative-fit-index, *SRMR* standardized root mean square residual, *RMSEA (CI*) root mean square error of approximation (confidence interval), *TLI* Tucker-Lewis Index, *GFI* Goodness-of-Fit Index

Furthermore, the invariance of the one-factor model was tested for the whole sample across gender and six age groups. Results of the measurement invariance tests are shown in Table 5.

**Table 5:** Tests for invariance across gender and age groups for the whole study sample (*n* = 2.508)

	N	χ ^2^ (df)	Δ χ ^2^	Δ *p*	CMIN/DF	CFI	Δ CFI	RMSEA	Δ RMSEA
Gender									
Men	1.171	28.508 (2)			14.254	.982		.106	
Women	1.330	16.831 (2)			8.415	.989		.075	
Multigroup analysis									
Configural model		45.340 (4)			11.335	.986		.064	
Metric model		53.155 (8)	7.815	.099	6.644	.984	.002	.048	.016
Scalar model		82.542 (12)	29.388	<.001	6.879	.975	.009	.049	.001
Age									
14–29 years	444	0.209 (2)			0.104	1		0	
30–39 years	330	2.908 (2)			1.454	.998		.037	
40–49 years	447	29.243 (2)			14.621	.950		.175	
50–59 years	466	10.147 (2)			5.073	.985		.094	
60–69 years	393	16.285 (2)			8.142	.957		.135	
≥ 70 years	421	8.740 (2)			4.370	.986		.090	
Multigroup analysis for all age groups									
Configural model		67.528 (12)			5.627	.980		.043	
Metric model		96.920 (32)	29.393	.080	3.029	.977	.003	.029	.014
Scalar model		239.290 (52)	142.370	<.001	4.602	.933	.044	.038	.009
Multigroup analysis for all age groups except 14–29 years and ≥70 years									
Configural model		58.577 (8)			7.322	.972		.062	
Metric model		73.271 (20)	14.694	.259	3.664	.971	.001	.040	.022
Scalar model		101.076 (32)	27.805	.006	3.159	.962	.009	.036	.004

As the index of ΔCFI indicates, this model can be assumed metric and scalar invariant across males and females. Regarding the invariance tests across six different age groups metric invariance can be confirmed, but due to ΔCFI > .01 scalar invariance could not be confirmed. Following the procedure of Gregorich (2006) to test for partial invariance did not reduce ΔCFI smaller than .01. Exploring the intercepts of all age groups revealed that the age groups of 14–29 years and ≥70 years had lower intercepts in all items except for item 3 compared to all other age groups (data not shown). Therefore, measurement invariance was tested again for all age groups except 14–29 years and ≥70 years. Results clearly indicate metric and scalar invariance across these four age groups (Table 5).

### Internal consistency

The parameter of internal consistency (Cronbach’s α) for the BRCS scale reached the value of α =0.78.

### Normative data

Table 6 summarizes the normative data for the different age levels and both genders. Percentiles from this table can be used to compare an individual subject’s BRCS score with those determined from the general population reference group based on age and gender.

**Table 6:** Normative data from the general population for the BRCS

Total		Men							Women						
	14–92 y *N* = 2508	14–24 *n* = 134	25–34 *n* = 152	35–44 *n* = 180	45–54 *n* = 213	55–64 *n* = 225	65–74 *n* = 177	≥75 *n* = 93	14–24 *n* = 123	25–34 *n* = 208	35–44 *n* = 202	45–54 *n* = 232	55–64 *n* = 229	65–74 *n* = 204	≥75 *n* = 136
M															
S.D.															
Sum Score	Percentile^a^														
4	0.2	1.1	.7	.6	0.7	–	–	–	.8	.5	.5	–	–	–	1.1
5	0.6	–	–	–	–	.7	.6	–	–	1.2	1.0	–	–	.5	–
6	0.9	2.7	–	–	–	–	–	–	1.6	–	1.5	.4	–	–	2.2
7	1.5	–	1.3	2.2	1.42	2.0	1.4	2.3	–	2.5	–	1.1	–	1.7	3.7
8	3.0	4.6	3.0	4.4	2.1	3.4	3.4	5.1	3.3	3.9	3.0	2.0	.7	3.7	5.6
9	4.9	6.8	4.9	5.8	5.0	4.5	5.4	9.6	6.2	5.6	5.2	3.0	1.3	5.7	8.9
10	8.2	11.4	6.9	6.9	9.5	6.3	9.4	15.7	9.0	7.8	8.0	4.6	2.4	11.8	17.8
11	12.7	16.3	10.2	9.7	13.0	8.7	14.5	19.7	15.2	10.5	12.9	9.1	6.3	20.0	27.4
12	19.5	23.1	17.4	15.3	18.7	13.4	19.3	27.5	24.2	17.2	18.4	16.5	14.4	26.9	39.3
13	27.9	33.7	26.0	21.1	26.8	20.1	28.1	39.3	32.4	27.2	24.6	23.2	25.6	33.7	53.3
14	38.0	44.7	35.5	30.0	37.2	28.8	40.6	49.4	42.6	39.0	34.8	31.4	34.5	42.9	64.8
15	49.4	54.6	46.7	41.7	48.1	40.4	51.1	59.6	51.2	51.2	49.0	43.3	47.2	55.2	72.6
16	62.4	69.3	57.2	52.5	58.5	56.3	63.4	72.5	62.3	64.5	63.4	60.4	61.1	67.5	81.5
17	75.7	83.3	69.4	65.3	72.3	72.8	77.3	82.0	77.5	77.2	74.4	76.0	75.3	79.3	90.4
18	84.6	88.6	79.6	76.9	83.4	84.2	85.5	84.8	87.7	85.1	83.6	85.3	84.1	88.4	94.4
19	90.3	92.4	87.2	83.9	88.6	90.2	91.8	87.6	92.2	89.5	91.3	92.6	89.1	93.8	97.8
20	96.3	97.4	95.7	93.3	95.3	96.0	97.7	94.5	96.7	95.6	97.3	98.1	95.6	98.0	99.6

^a^Percentiles indicate the rank of the subject compared to other subjects of the same age group and gender

## Discussion

Regarding the factor structure of the BRCS, results of EFA and CFA clearly support the assumption of the BRCS to be unidimensional, representing one latent factor. On the subject of the measurement invariance test, metric and scalar invariance across males and females could be confirmed. Concerning the six different age groups examined in the present study, metric invariance could be confirmed, but scalar invariance could not be confirmed across all groups. Thus, from a statistical perspective, mean comparisons including persons aged 14–29 as well as ≥  70 years and persons aged 30–69 should be interpreted with caution. Overall, in studies that used resilience scales, age effects turned up when the samples had a broader age range and they were less likely to turn up in samples of a narrower age range [23, 24].

An additional main result of this study was the standardization of the BRCS with the provision of normative data from the general population for different age and gender groups. Given that age and gender specific comparative data were generated based on subgroups consisting of *N* = 93 to *N* = 232 subjects each, the sample sizes were sufficient to compute normative data. Resilience scores varied according to gender, similar to other recent studies [25, 26], yet the effect size was small, likewise reported elsewhere [24, 27].

The obtained findings could be further utilized as reference categories in community studies and health care settings [1, 4]. For the communities, promotion of resilience gains more and more significant importance in terms of a healthy, well-educated population [28]. A potential limitation of this general population study is that it is a cross-sectional study which does not allow for interpretations of causality or possible mediator effects. Further longitudinal evaluations of the BRCS are necessary to demonstrate its performance also in different clinical target populations.

With the present study that assesses the BRCS in a representative sample of the general population, this instrument can be assumed to have good internal consistency and the provision of norm values allow comparing the results of further studies with age and gender specific norms of the general population.

## Conclusion

The normative data provide a framework for the interpretation and comparisons of resilient coping with other populations. Results demonstrate a special importance of age in the understanding of resilience.

## Additional file

Additional file 1: SPSS file of BRCS data (N=2508). (SAV 187 kb)

## Abbreviation

BRCS: Brief Resilient Coping Scale

## References

[1] Luthar SS, Cicchetti D, Becker B (2000). The construct of resilience: a critical evaluation and guidelines for future work. Child Dev.

[2] Rutter M (1987). Psychosocial resilience and protective mechanisms. Am J Orthop.

[3] Southwick SM, Bonanno GA, Masten AS, Panter-Brick C, Yehuda R. Resilience definitions, theory, and challenges: interdisciplinary perspectives. Eur J Psychotraumatol. 2014;5:369–77. 10.3402/ejpt.v5.25338 PMC4185134 25317257

[4] Rutter M (2013). Annual research review: resilience--clinical implications. J Child Psychol Psychiatry.

[5] Mohaupt S (2009). Review article: resilience and social exclusion. Social Policy & Society.

[6] Masten A, Powell J, Luthar S (2003). A resilience framework for research, policy and practice. Resilience and vulnerability: adaptation in the context of childhood adversities.

[7] Sinclair VG, Wallston KA (2004). The development and psychometric evaluation of the brief resilient coping scale. Assessment.

[8] Heinen I, Bullinger M, Kocalevent RD (2017). Perceived stress in first year medical students - associations with personal resources and emotional distress. BMC Med Educ.

[9] Lopez-Pina JA, Meseguer-Henarejos AB, Gascon-Canovas JJ, Navarro-Villalba DJ, Sinclair VG, Wallston KA (2016). Measurement properties of the brief resilient coping scale in patients with systemic lupus erythematosus using rasch analysis. Health Qual Life Outcomes.

[10] Rice V, Liu B (2016). Personal resilience and coping part II: identifying resilience and coping among U.S. military service members and veterans with implications for work. Work.

[11] Edo-Gual M, Monforte-Royo C, Aradilla-Herrero A, Tomas-Sabado J (2015). Death attitudes and positive coping in Spanish nursing undergraduates: a cross-sectional and correlational study. J Clin Nurs.

[12] Cosco TD, Kaushal A, Richards M, KUH D, Stafford M (2016). Resilience measurement in later life: a systematic review and psychometric analysis. Health Qual Life Outcomes.

[13] Beutel ME, Tibubos AN, Klein EM, Schmutzer G, Reiner I, Kocalevent RD, BRAHLER E (2017). Childhood adversities and distress - the role of resilience in a representative sample. PLoS One.

[14] Kocalevent RD, Mierke A, Brähler E, Klapp BF, Kemper CJ, Brähler E, Zenger M (2014). BRCS brief resilient coping scale. Psychologische und sozial-wissenschaftliche Kurzskalen.

[15] Hedges L, Olkin I (1985). Statistical methods for meta analysis.

[16] Velicer WF. Determining the number of components from the matrix of partial correlations. Psychometrika. 1976;41(3):321–7.

[17] O'Connor BP. SPSS and SAS programs for determining the number of components using parallel analysis and Velicer's MAP test. Behavior Research Methods, Instrumentation, and Computers. 2000;32:396–402. 10.3758/bf03200807 11029811

[18] Schermelleh-Engel K, Moosbrugger H, Müller H. Evaluating the Fit of Structural Equation Models: Tests of Significance and Descriptive Goodness-of-Fit Measures. Methods of Psychological Research. 2003;8(2):23–74.

[19] Gregorich SE. Do self-report instruments allow meaningful comparisons across diverse population groups? Testing measurement invariance using the confirmatory factor analysis framework. Medical Care. 2006;44(11 Suppl 3):S78–94. 10.1097/01.mlr.0000245454.12228.8f PMC1808350 17060839

[20] Cheung G, Rensvold R. Evaluating Goodness-of-Fit Indexes for Testing Measurement Invariance. Journal Structural Equation Modeling: A Multidisciplinary Journal. 2002;9(2):2000.

[21] Little TD, Slegers DW, Card NA. A Non-arbitrary Method of Identifying and Scaling Latent Variables in SEM and MACS Model. Structural Equation Modeling. 2006;13(1);59–72.

[22] Crawford JR, Garthwaite PH, Lawrie CJ, Henry JD, Macdonald MA, Sutherland J, Sinha P (2009). A convenient method of obtaining percentile norms and accompanying interval estimates for self-report mood scales (DASS, DASS-21, HADS, PANAS, and sAD). Br J Clin Psychol.

[23] Ahern NR, Kiehl EM, Sole ML, Byers J (2006). A review of instruments measuring resilience. Issues Compr Pediatr Nurs.

[24] Schumacher J, Leppert K, Gunzelmann T, Strauß B, Brähler E (2005). Die Resilienzskala - Ein Fragebogen zur Erfassung der psychischen Widerstandsfähigkeit als Personmerkmal. Z Klin Psychol Psychiatr Psychother.

[25] Liebenberg L, Ungar M, Van de Vijver F (2012). Validation of the child and youth resilience Measure-28 (CYRM-28) among Canadian youth. Res Soc Work Pract.

[26] Merrell KW, Felver-Gant JC, Tom KM (2011). Development and validation of a parent report measure for assessing social-emotional competencies of children and adolescents. J Child Fam Stud.

[27] Eisenhart-Rothe A, Zenger M, Lacruz ME, Emeny R, Baumert J, Haefner S, Ladwig KH (2013). Validation and development of a shorter version of the resilience scale RS-11: results from the population-based KORA-age study. BMC Psychol.

[28] Castleden M, Mckee M, Murray V, Leonardi G (2011). Resilience thinking in health protection. J Public Health (Oxf).

